# Correction: Fabris et al. Exploring Multivariate Profiles of Psychological Distress and Empathy in Early Adolescent Victims, Bullies, and Bystanders Involved in Cyberbullying Episodes. *Int. J. Environ. Res. Public Health* 2022, *19*, 9871

**DOI:** 10.3390/ijerph21030297

**Published:** 2024-03-04

**Authors:** Matteo Angelo Fabris, Claudio Longobardi, Rosalba Morese, Davide Marengo

**Affiliations:** 1 Department of Psychology, University of Turin, Via Verdi 10, 10124 Turin, Italy; 2 Faculty of Communication, Culture and Society, Università della Svizzera italiana, Via Buffi 13, 6900 Lugano, Switzerland; 3 Faculty of Biomedical Sciences, Università della Svizzera italiana, Via Buffi 13, 6900 Lugano, Switzerland

## Missing Corresponding

In the published publication [1], there was an error regarding the corresponding authors. Due to an error, Rosalba Morese, who was responsible for the manuscript during the submission and production process, was not added as corresponding author in the original paper. This correction has been approved by the Academic Editor. The original publication has also been updated.
